# Recent Advances in Subunit Vaccine Carriers

**DOI:** 10.3390/vaccines4020012

**Published:** 2016-04-19

**Authors:** Abhishek Vartak, Steven J. Sucheck

**Affiliations:** Department of Chemistry and Biochemistry, The University of Toledo, 2801 West Bancroft Street, Toledo, OH 43606, USA; Abhishek.Vartak@rockets.utoledo.edu

**Keywords:** nanoparticles, vaccines, liposomes, polymeric nanoparticles

## Abstract

The lower immunogenicity of synthetic subunit antigens, compared to live attenuated vaccines, is being addressed with improved vaccine carriers. Recent reports indicate that the physio-chemical properties of these carriers can be altered to achieve optimal antigen presentation, endosomal escape, particle bio-distribution, and cellular trafficking. The carriers can be modified with various antigens and ligands for dendritic cells targeting. They can also be modified with adjuvants, either covalently or entrapped in the matrix, to improve cellular and humoral immune responses against the antigen. As a result, these multi-functional carrier systems are being explored for use in active immunotherapy against cancer and infectious diseases. Advancing technology, improved analytical methods, and use of computational methodology have also contributed to the development of subunit vaccine carriers. This review details recent breakthroughs in the design of nano-particulate vaccine carriers, including liposomes, polymeric nanoparticles, and inorganic nanoparticles.

## 1. Introduction

The introduction of vaccines to prevent infectious disease has had a transformational effect on human health. For example, the declaration of the global eradication of wild poliovirus type 2 was one of the highlights of the Global Commission for the Certification of Poliomyelitis Eradication (GCC) meeting in September 2015. Further, vaccination against measles, a highly contagious disease caused by morbillivirus in the paramyxovirus family has resulted in a 79% decrease in measles deaths globally from 2000 to 2014. Another example was the introduction of a meningitis A conjugate vaccine, which expedited the near elimination of the deadly disease in the African “meningitis belt”. These stunning accomplishments emphasize the importance and need for vaccines that profoundly contribute to well-being of society.

A vaccine can be generally defined as a biological preparation that contributes to active immunity against a particular disease. The pioneering work by Edward Jenner for the smallpox vaccination, followed by Louis Pasteur for an anthrax vaccine, lead to development of vaccines against many other infectious diseases. The World Health Organization (WHO) has reported vaccines for the prevention and control of 27 infections, with many other vaccines in the discovery pipeline. A candidate vaccine against malaria (RTS,S) recently completed phase III clinical trials involving approximately 15,000 infants and young children in seven sub-Saharan African countries. Two Ebola candidate vaccines are also in the final stages of clinical trials. The research community is also making sustained efforts toward the development of vaccines against diseases which are difficult to treat, such as cancer [1,2], hepatitis C, and tuberculosis [3,4,5].

## 2. Types of Vaccines

Vaccination involves exposure of an antigen, derived from a disease-causing agent, to the immune system with the aim to develop active immunity against the antigen. When the vaccinated individual comes in contact with the causative micro-organism, a strong protective immune response occurs. The optimal properties of any vaccine include long-lasting immunity, lack of autoimmunity or hypersensitivity, ease of administration, and storage. Additionally, vaccine components should be safe, and, specifically, the vaccine itself should not result in the disease state.

The different types of vaccines (Table 1) include live attenuated vaccines, which consist of live, weakened, or modified disease causing micro-organisms, which results in a limited infection that is sufficient to invoke immune response, but not long-lived enough to cause the actual disease state [6,7]. The attenuation is achieved by repeated culturing of disease causing agent in a foreign host. The less virulent mutant adapted to the foreign host can be used for vaccination. Killed/Inactivated vaccines work with help of different chemical methods, radiation, or heat. The pathogen is inactivated so it cannot replicate in the host and is used as the vaccinating agent [6,7]. Bacterial vaccines have generally used dead micro-organisms, while viral vaccines are composed of inactivated agents. Recombinant/DNA are experimental vaccines composed of genes encoding antigen inserted into a vector (recombinant vaccine) [6,7]. The DNA can be injected or attached to a carrier, such as metallic nanoparticles (DNA vaccine). *In situ* generation of antigenic protein can lead to strong immune response. Subunit vaccines contain a purified antigen instead of using whole microorganisms. Purified antigens could be toxoid, subcellular fragment, or surface molecules, which are transported by different carriers [6,7]. Immune response to subunit vaccine differs based on the antigen used. Protein antigens usually give rise to T-cell dependent adaptive immune response while polysaccharide antigens generate T-cell independent response. Conjugated vaccines can be defined as subclass of subunit vaccines as protein carriers are used to carry polysaccharide based antigen.

## 3. Immunology of Vaccines

Immunology is a science that studies the structure and function of the immune system, which itself is sub-classified into innate and adaptive immune systems. The innate immune system is non-specific and quickly forms the body’s first line of defense. Phagocytes, soluble peptides, and proteins are complement molecules, while natural killer (NK) cells are key elements of innate immune system. On the other hand, the adaptive immune system gives a pathogen or antigen a specific response along with immunological memory. The adaptive response takes days to weeks to develop. T lymphocytes and B lymphocytes are essential for the adaptive immune response.

Dendritic cells (DCs) play important roles in both innate as well as adaptive immunity. These antigen presenting cells (APCs) are the messengers that call for help from the adaptive immune response when an infection outruns innate immunity [8]. The immature dendritic cells function as phagocytes in innate immunity, participating in capture, uptake, and processing of antigen. While moving to secondary lymphoid tissue, these cells gain capacity to activate naïve T cells (CD4^+^ and CD8^+^). These APCs stop phagocytosis and present antigen on their surfaces with a high density during this migration. Such cells in lymph nodes are called mature or activated dendritic cells.

Activation of naïve T cells requires binding antigen to specific T cell receptors (TCR), along with a co-stimulatory signal. The receptor responsible for the co-stimulatory signal is called CD-28 and presents on T cells that bind to the B7 ligand on dendritic cells. Proliferation and differentiation of naïve T cells into effector cells is initiated by these intracellular signals. CD8^+^ T cells or CD4^+^ T cells form based on antigen presentation by either MHC-class I or MHC-class II molecules, respectively, on dendritic cells. In most cases, extracellular antigens are presented by MHC-class II on APCs, which in turns activate CD4^+^ T cells, also known as T helper cells. On the other hand, intracellular antigens presented by MHC-class I molecules on APCs lead to the activation of CD8^+^ T cells, also known as cytotoxic T cells.

Although the above-mentioned distinction is not absolute, the phenomenon of presentation of antigen located in the MHC-class II pathway to MHC-class I pathway is called “cross presentation”. Such presentation helps to develop both CD4^+^, as well as CD8^+^ response, against antigens. The understanding of cross-presentation is important to the success of producing an effective immune response to a vaccine [9]. There is a major focus on increasing cross-presentation of antigen in DCs targeting vaccines.

In the case of subunit vaccine preparation, polysaccharide antigens are poorly immunogenic. Therefore, immuno-adjuvants are added along with antigen in the vaccine carrier to augment the immune response. Another approach is to conjugate bacterial proteins to these antigens, as mentioned earlier. Immuno-adjuvants, especially Toll-like receptor (TLR) ligands are increasingly used in vaccine design [10,11]. Signals from TLRs on dendritic cells improve the processing and presentation by APCs. They also play a role in maturation of dendritic cells.

## 4. Minimal Subunit Vaccine Development

The classical approach of conjugating carbohydrate antigens to a protein, such as bovine serum albumin (BSA) and keyhole limpet hemocyanin (KLH), is reviewed elsewhere [12,13]. The problems associated with these conjugated vaccines include: Variation in antigen loading, immunogenic linkers, and antigenic carrier proteins lead to the development of minimal subunit vaccines. Research on these vaccines is happening in three areas: Minimal-peptide antigens, improved immune-adjuvant and the use of novel vaccine carriers.

Single molecule two-component vaccines with monomeric, trimeric tumor-associated carbohydrate antigens (TACAs), along with TLR ligands were developed [14,15,16,17,18]. To facilitate antibody class switching from IgM to IgG, a helper T-cell epitope was incorporated into a two-component vaccine to obtain a fully synthetic multi-component vaccine, including phospholipid-based liposomes [19,20], which serves as the carrier. Several polysaccharide antigens specific to cancer [21,22,23,24,25], HIV [26], and other infections [25,27], have been identified and incorporated into this type of vaccine. Investigating new immuno-adjuvants with good safety profiles is another way to potentially advance this platform. Adjuvants can enhancing antigen presentation through dendritic cell (DC) maturation, produce a depot effect, or induce APCs to release cytokines [28,29,30]. Different types of adjuvants, such as bacterial, gel-type, emulsifier, and synthetic, are known [31].

In recent years, advancement of vaccines has focused on one major area; *i.e.*, vaccine carriers [32,33,34,35]. Liposomes, archeosomes, virosomes, virus-like particles (VLPs), polymeric, and inorganic micro-, nano-particles have all been developed. Some of these particulate carriers show adjuvant properties as well. Herein, we present recent case studies of advancements in these vaccine carriers with their physio-chemical attributes.

## 5. Liposomes

**Figure 1:**
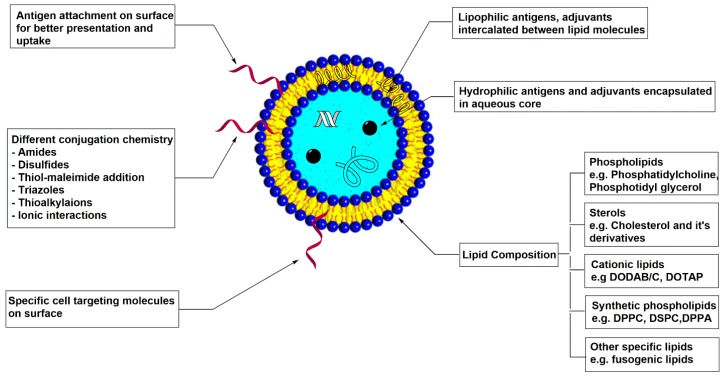
Variations with liposomal vaccine delivery system.

Liposomes are biocompatible, completely biodegradable, self-assembled vesicular structures composed of lipid bilayers. Liposomes have been extensively used as drug delivery vehicles for years, but, in last decade, their use as a vaccine carrier has increased (Figure 1). Considering their wide applications, liposomes have been extensively reviewed by many groups [36,37,38,39,40]. These reviews cover methods of preparation, physio-chemical properties, such as particle size, charge, lamellarity, and their effects on drug or vaccine delivery and different conjugation chemistry.

Liposomes are advantageous over other vaccine carriers due to their tolerability by the human body and lack of toxicity, along with their chemical and structural flexibility. Chemical flexibility refers to the ability of the liposome to encapsulate either a hydrophilic antigen or adjuvant or a lipophilic component, which can intercalate between the lipid molecules [41]. Surface conjugation of hydrophilic antigens is also possible, which facilitates a better uptake by phagocytes because of better accessibility of antigen [42,43,44,45,46]. Structural flexibility refers to the tuning of liposomal properties by modifying lipid composition. For example, the use of cationic lipids has increased, as cationic liposomes are known to improve cytosolic release of antigens by affecting endosomal membrane integrity [47,48,49,50].

There are different methods of preparation of liposomes, such as physical dispersion, solvent dispersion, and detergent solubilization, which are reviewed elsewhere [51,52,53,54]. The general procedure for liposomal formulation involves dissolution of lipid content in an organic solvent. The organic solvent is then evaporated to obtain a thin film of lipid. After drying, the film is hydrated with an aqueous system containing hydrophilic antigen and adjuvants. The resulting vesicular structures are then subjected to freeze-thaw cycles, sonication, or membrane extrusion to ensure entrapment efficiency, size, and lamellarity according to application. In the case of hydrophobic components, they are dissolved in organic solvents along with lipids.

### 5.1. Physiochemical Properties

Recent advances in physiochemical properties, namely particle size, fusogenicity, and lipid compositions of liposomal vaccine carriers, are discussed briefly.

Particle size: This important property of a vaccine vehicle governs cellular trafficking to secondary lymph nodes, antigen uptake, and cellular responses. Monolova and co-workers studied the kinetics of trafficking of small *versus* large Virus Like Particles (VLPs) [55]. They observed free drainage of small particles (20–200 nm) towards lymph nodes (LN), while large particles (500, 1000 nm) are dependent on dendritic cells (DCs) for transport to LN. Several experiments have been performed to evaluate the effect of liposomal size on T_H_1 and T_H_2 responses [56].

Fusogenicity: Ability to fuse with the plasma membrane or endosomal membrane, *i.e.*, fusogenicity has been widely exploited. Greater cytoplasmic delivery of extracellular antigen elicits a higher cellular immune response. Miyabe and co-workers recently discovered a new class of adjuvant, cyclic di-GMP, which induces the production of type I interferons that can enhance immuno-stimulatory activity [57]. They also reported the use of the pH-sensitive and highly fusogenic synthetic lipid, YSK05. During the comparative study of cationic liposomes, commercially-available transfection reagent and their optimized lipid composition with YSK05, they observed higher INF-β (pg/ml) production with the YSK05 fusogenic lipid. Increased expression of CD80 and CD86, as well as higher CTL activity, suggests the importance of fusogenicity.

Lipid composition: The effect of selection of lipids on liposomal properties, such as particle size, stability, and ability of inducing maturation of DCs, is a prerequisite in order to achieve an optimized vaccine carrier. The effect of lipid composition on membrane fluidity has been studied using gel-liquid transition temperature and membrane phase behavior experiments [58,59,60,61,62]. The selection of a lipid determines membrane fluidity, which is linked to cellular trafficking and antigen presentation to APCs. In addition, the depot effect of an antigen at the site of injection is based on the choice of lipid. This effect has been studied by Christensen, *et al.* [63]. The effect of degree of lipid saturation on T_H_1-directed immune response was observed with rigid, saturated dimethyl dioctadecyl ammonium (DDA) lipid and fluid, unsaturated dimethyl dioleoyl ammonium (DODA) lipid. More than 100 times the priming ability and an elevated level of co-stimulatory molecules was observed with rigid lipids, which was correlated to higher retention capacity of lipids. A DoE (design of experiments) approach was used by Soema, *et al*. to study effect of different lipids such as Egg phosphatidylcholine (EPC), 1,2-dioleoyl-sn-glycero-3-phosphoethanolamine (DOPE), 1, 2-dioleoyl-3-trimethylammonium propane (DOTAP) and 3beta-[N-(N′,N′-dimethylaminoethane) carbamoyl] cholesterol (DC-Chol) on DC maturation [64]. They observed that the liposomes containing DOTAP lipids were able to induce DC maturation and had a higher zeta potential value, which dictates the colloidal stability of the formulation.

### 5.2. Liposome-Polymer Hybrid Vaccine Carrier

Recently, the use of cationic liposomes has increased owing to their higher internalization by macrophages and dendritic cells compared to neutral, as well as anionic liposomes. Electrostatic interaction with the negatively-charged plasma membrane and the ability to disrupt endosomal membrane are beneficial for higher immune-stimulatory response [65].

The major challenge of cytotoxicity of these cationic liposomes at high concentration was addressed by Fan, *et al*. [66]. Cationic DOTAP liposomes were surface-modified using thiolated hyaluronic acid (HA) biodegradable polymer with ionic complexation. Liposome-HA particles were coated with PEG (polyethylene glycol) using dithiol conjugation in the presence of an oxidizing agent, chloroamine T, for better stability and steady antigen release. The optimal concentration of HA was decided based on fluorescence resonance energy transfer (FRET) analysis and effect on particle size of hybrid particle after ionic interaction. Enhanced biocompatibility as well as reduced cytotoxicity of hybrid particles compare to DOTAP liposomes was observed using bone marrow derived dendritic cells (BMDCs) with various concentrations. In all, liposomal polymeric nanoparticle based intranasal vaccine with F1-V (fusion protein of fraction 1 pilus and LcrV antigen from *Yersinia pestis*, causative agent of pneumonic plague) and MPLA as an adjuvant was discovered.

### 5.3. Dendritic Cells (DCs) Targeting Liposomal Vaccine Carrier

DCs are the leading antigen presenting cells as a result of their ability to cross-represent extra cellular antigen via MHC class I molecule and potency to promote T-cell proliferation. As a result, targeting DCs for enhanced immune response has been exploited with the help of different receptors expressed by DCs.

In recent studies, Karmakar, *et al.* observed improved antigen uptake and cellular response by targeting Fcγ receptors on DCs [67,68,69]. The natural abundance of specific natural anti-Rhamnose antibodies in the human population was exploited using L-rhamnose as a targeting ligand [70]. Indirect targeting was achieved via the presence of anti-Rhamnose antibodies, generated in mice, bound to the rhamnose ligand on the liposomal vaccine. Higher CD8^+^ T-cell specific INF-γ production was observed with targeting ligands on liposomes.

C-type lectin receptors (CLRs) on DCs were exploited by Jiang, *et al.* [71]. CLRs are known to bind to galactose, Lewis X mono or oligosaccharides, and N-acetylgalactosamine. Higher levels of pro-inflammatory cytokines with galactosylated liposomal vaccine were observed, verifying the significance of DC targeting to immunological response.

Due to the tailoring properties and versatility that can be achieved with liposomes, these carrier systems are under investigation for further developments in vaccine formulation. Recent advancements with liposomal vaccine delivery systems for different diseases are summarized in Table 2. About 39 clinical studies involving liposomes as vaccine carriers are listed on Clinical Trials.gov. Some of those are listed with their respective statuses in Table 3.

## 6. Polymeric Nanoparticles

**Figure 2:**
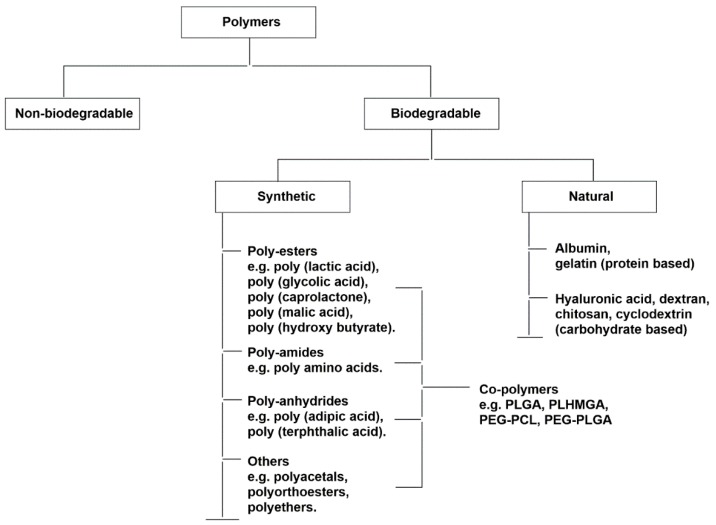
Polymers used in vaccine delivery.

Polymeric nanoparticles are also under investigation for their potential use as vaccine carrier platforms [83,84,85,86,87,88]. Apart from similar advantages, such as biocompatibility, safety, and flexibility of liposomal carrier systems, a wide variety of natural, as well as synthetic, bio-degradable polymers and block co-polymers create opportunities to tailor and improve these materials (Figure 2). By varying nanoparticle size, shape, surface charge, type of polymer, and polymer concentration, researchers can optimize polymeric nano-particulate carriers. Recent advances in these areas are discussed further.

### Physiochemical Properties

Particle size: Experimental data are available on particle size and its effects on drug distribution and respective immunological applications. Recently, Silva and co-workers evaluated micro- and nano-poly (lactic-co-glycolic acid) (PLGA) particles, their phagocytosis by DCs, and respective immune responses [89]. They observed superior antigen presentation with nano-PLGA particles than micro-particles with balanced T_H_1 and T_H_2 type antibody response compared to vaccination with incomplete Freund’s adjuvant (IFA). Another study, with hydrophilic polyester (poly(D,L lactic-co-hydroxy methyl glycolic acid) (pLHMGA)) nanoparticles carrying synthetic long protein derived from HPV 16 E7 onco-protein and TLR 3 ligand, showed comparable results with IFA formulation without any local adverse effects of IFA [90].

Particle shape and geometry: The importance of physical attributes, other than particle size, such as shape and elasticity, have not been comprehensively studied. However, recent studies suggest an impact of these other aspects on circulation time, phagocytosis, immune cell targeting, and cargo release. Kumar and co-workers synthesized polystyrene particles of different shapes and sizes to investigate their interactions with the immune system [91]. Spherical polystyrene particles (193 nm and 521 nm) were stretched by the film stretching method to obtain rod-shaped particles (376 nm and 1530 nm in length) containing a model antigen (ovalbumin). Smaller spherical nanoparticles (193 nm) produced higher antibody titers when compared to large spheres, but, interestingly, in the case of rod-shaped particles, the trend was reversed (high titers for 1530 nm than 376 nm). T_H_1 and T_H_2 biased immune response was observed to be based on shape, as spherical particles swayed the immune response to a strong T_H_1, while the rod-shaped particles shifted the response to T_H_2.

Cylindrical, 80 × 180 nm, hydroxy-poly (ethylene glycol) (PEG) was shown to elicit an improved immune response with sustained antigen presentation [92]. Hydrogel PEG particles were prepared using particle replication in nonwetting templates (PRINT) technology to achieve better control of NP size, charge, and surface functionality. Anionic cylindrical particles were observed to have better lymphatic drainage compare to other variable nanoparticles. These rod/cylindrical particles seem to be advantageous over traditional spherical polymeric particles owing to their higher cellular uptake, as well as antigen loading. A computational model for nanoparticle transport and distribution was studied by Tan and co-workers, which simulated the Brownian dynamics and fluid mechanics of nanoparticle in blood vessels [93].

Elasticity of the polymeric platform also plays a role in phagocytosis, which is crucial for antigen uptake and presentation. Anselmo and co-workers synthesized hydrogel nanoparticles composed of PEG diacrylate (PEGDA) via the nano-emulsion method [94]. The elastic modulus of the particle was controlled by volume fraction of PEGDA in the nano-emulsion. Hard nanoparticles were observed to be phagocytosed with a rate of 3.5-fold, or higher, compared to soft nanoparticles. Long circulation of both types of nanoparticles were attributed to the presence of PEG, which provides stability to particles by avoiding opsonization, reticuloendothelial system (RES) clearance, and decreased interactions with the extracellular matrix (ECM).

The significance of cellular and/or humoral response against any antigen is well established. The intracellular fate of the antigen decides the induction of an antigen-specific immune response. Antigens present in endosomes lead to specific humoral immune responses, while endosomal escape of antigens, followed by proteosomal processing, leads to the specific cellular immune response. The effect of hydrophobicity of polymers on endosomal escape and their membrane interactions have started to gain attention. Shima and co-workers modified the hydrophilic backbone of poly (γ-glutamic acid) (γ-PGA) with hydrophobic L-phenylalanine ethyl ester (L-Phe) with different degrees of grafting [95]. The membrane disruptive property of γ-PGA-Phe nanoparticles was observed to be dependent on surface hydrophobicity. It can be concluded that the balance between hydrophilicity and hydrophobicity is important for cytosolic delivery of antigens, which could be polymer specific. The role of hydrophobicity in manipulating the rate of polymer degradation is also well known. In the case of the PLGA polymer, a higher ratio of hydrophilic monomer component, *i.e.*, glycolic acid, causes an increased rate of degradation.

PLGA nanoparticles: Among all natural, as well as synthetic, bio-degradable polymers, PLGA has been extensively used for vaccine delivery. PLGA is an Food and Drug Administration (FDA)-approved aliphatic co-polymer, composed of varying degrees of lactic acid and glycolic acid monomers. As mentioned earlier, PLGA nanoparticles with various antigens are capable of inducing a stronger CD8^+^ T-cell immune response compare to soluble antigen. There are several reports of the chemical modifications and degradation of antigens loaded in PLGA particles, exposing some of the drawbacks of the carrier. However, PLGA continues to be used as a vaccine carrier. Some recent reports of this particulate carrier with different modification are mentioned in Table 4.

PLGA co-polymers, along with other co-polymers, are being investigated for different aspects of vaccinology to obtain an improved immune response. Higher surface conjugation, as well as physical adsorption of ovalbumin (OVA) on maleic anhydride (MA) grafted poly (lactic acid) (PLA) (PLA-g-MA) [104], compared to only PLA, emphasizes the untapped potential and scope of use for co-polymers in this area. To overcome plausible flaws in PLGA particles, pLHMGA particles were developed and shown to exhibit a cellular response with sustained release of antigen [105]. Kunda and co-workers developed a dry powder inhalation formulation for pneumonia with poly (glycerol adipate-co-ω-pentadecalactone) (PGA-co-PDL) nano-particles and L-leucine micro-particles to avoid exhalation of the nanoparticle because of low inertia [106].

## 7. Inorganic Nanoparticles

Recently, Zhang and co-workers presented promising CD8^+^ T cell results with polyelectrolyte multilayers (PEM) assembled on gold nanoparticles (AuNPs) [107]. PEMs are self-assembled structures constructed via layer-by-layer (LbL) deposition using electrostatic interactions between positively and negatively charged electrolytes. Positively charged antigen and negatively charged immuno-adjuvant on gold nanoparticles resulted in a new vaccine platform. Greater control over antigen loading is a key advantage in vaccine design. The concept of PEMs was further exploited by Chiu, *et al.* to develop vaccine capsules made up of PEMs without any vehicle [108]. Calcium carbonate was used as solid support for PEM deposition, which was later removed.

The ease of synthesis of these inorganic nanoparticles, with precise control over mono-dispersity, size and shape, higher cargo loading, and colloidal stability, outweigh some limitations, such as their non-biodegradability. Recently, AuNPs have been used in immunotherapy as they are inert and can be easily functionalized with desired molecules. Chiodo, *et al.* synthesized AuNPs with tetra- and pentamannosides in order to mimic clusters of HIV gp120 [109]. Similar efforts have been made to develop AuNP-based vaccines for cancer [110,111,112,113], influenza [114], malaria [115], FMD [116], and HIV [117], by conjugating respective antigens on the surface. Sungsuwan, *et al.* developed lipid-coated iron oxide nanaoparticles with mucin-1 (MUC-1) antigens for cancer therapy [118]. The antigen-modified lipid was used to coat iron oxide nanoparticles to bind antigens on the surface without covalent modification. Meningitis A capsular polysaccharide fragments carrying iron oxide particle have been recently developed by Ramella, *et al*. [119]. Carbon nanoparticles, carbon nanotubes, silica, as well as calcium phosphate nanoparticles, are also under investigation for vaccine development.

The optical and photothermal properties of inorganic particles have been exploited in drug delivery as a tumor imaging and targeting tool. Based on recent studies, these treatments can have applications in immune therapy [120,121,122]. Heat shock proteins and tumor antigens released from dying tumor cells can activate the immune system. Koboyashi, *et al.* observed anti-tumor immunity via magnetic-nanoparticle-induced hyperthermia [123]. Application of an external magnetic field to the targeted magnetic nanoparticles increases tumor cells temperature without causing any harm to normal cells. Along with expected tumor cell death, the treatment resulted in an unexpected tumor specific response. These observations could lead to treatment addressing disease states, as well as immunotherapy.

## 8. Conclusions and Future Directions

This review summarizes recent developments in particulate carries for subunit vaccines. Development of a wide variety of nanoparticles with target specific modifications has a profound impact on the efficacy of vaccines. Understanding the fluid dynamics of these carriers, based on their physio-chemical variations, is the key to a better bio-distribution and antigen presentation. With advances in technology, investigators can achieve better control over the synthetic parameters of these vehicles to ensure reduced toxicity, antigen stability, and enhanced immunogenicity.

With the availability of new multifunctional carriers, the possibilities for novel vaccines have been greatly expanded. Comparison of different adjuvant loadings may be more accessible with these newer material. The synergistic effects of multiple immuno adjuvants may be addressed with DoE and simple conjugation chemistry to these nano-carrier. Parallel immune response studies of different nano-carriers, having the same antigen loading and adjuvant, could be interesting and may help in further development of hybrid nano-particles. In conclusion, recent studies highlighted in this review represent a step forward in dealing with current challenges in vaccinology and present new directions for future vaccine design.

## Figures and Tables

**Table 1:** Advantages and disadvantages of different vaccine types.

Vaccine Type	Advantages	Disadvantages
Live attenuated	A single dose of this type of vaccine is more potent as infectious agent can replicate in host.	May cause disease itself.
	Multiple doses may not be required.	Since vaccine is composed of live organism, storage is very critical.
	Since micro-organism itself is used, immune response against all antigens is generated.	Cannot be given to immunosuppressed individuals.
Killed/Inactivated	Safe to use in immunosuppressed patients.	Less immunogenic than live attenuated vaccines.
	Can’t cause disease state.	May require more booster doses to achieve desired immunity.
	Storage conditions are not critical compare to live attenuated vaccines.	
Recombinant/DNA	Better stability compare to traditional vaccines.	High production cost compare to other vaccine types.
	Storage conditions not critical.	Mutation in host DNA is possible in case of DNA vaccines.
	Better control on vaccine design as desired gene can be added or deleted.	
Subunit	Safe to use in immunosuppressed patients.	Less immunogenic than live attenuated vaccines.
	Cannot cause disease state.	Particular antigen or antigens should be identified causing the disease.
	Because of the purified antigenic component, less chances of side-effects.	
Conjugated	Safe to use in immunosuppressed patients.	Conjugation chemistry is difficult to control which could cause batch-wise variation.
	Cannot cause disease state.	Choice of carrier protein is crucial as they could be immunogenic causing suppression of antigenic immune response.
	Because of the purified antigenic component, less chances of side-effects.	

**Table 2:** Recent advancements in liposomal vaccine carrier system.

Disease	Lipid composition	Antigen	Adjuvant	Ref.
Pneumonic Plague	DOTAP, DOPE	Ovalbumin (OVA) F1-V recombinant fusion protein of *Y. pestis*	MPLA	[66]
Hepatitis B	SPC, MPC, SA	HBsAg	MPLA	[72]
Tuberculosis	DDA	BCG	TDB	[73]
	Yeast lipids	Alpha crystalline protein 1 (Acr 1)	-	[74]
HIV	DMPC, DMPG, Chol, and MPLA (ALF liposomes)	CN54 gp140 protein	MPLA,QS21 (a triterpenoid glycoside saponin)	[75]
	DOPC, DOPG, DSPE-PEG	MPER peptide	LACK-1 and HIV-30 (CD4^+^ epitope)	[76]
Cancer	DOTAP, DOPE, PC, DSPE-PEG	OVA-peptide, TRP-2peptide	Alpha-galactoceramide (α-GC)	[77]
	EYPC, DOPE, 3-Methyl glutarylated poly(glycidol) (MGluPG) and 3-methylglutarylated dextran	OVA	IFN-g-encoding plasmid DNA	[78]
	EPC, DSPE-PEG, Cholesterol, Sterylated R8	-	Alpha-galactoceramide (α-GC)	[79]
	POPE, YSK05, Cholesterol, DMG-PEG	-	Cyclic diGMP	[57]
	PC, Cholesterol, (α and β) Galactosyl-DLPE	OVA		[71]
	DPPC, Cholesterol, Rha-TEG-Cholesterol	Tn	Pam3Cys	[67]
DNA vaccine	EPC, Cholesterol, DSPE-PEG	IVTT mix , plasmid DNA (β galactosidase)	IVTT	[80]
Alzheimer’s disease	POPG, DOPC, Cholesterol, S1P	Amyloid-beta peptide (A β)	CFA/IFA	[81]
Foot and Mouth Disease (FMD)	Lecithin, Cholesterol	Inactivated FMDV	polyinosinic–polycytidylic acid (poly I:C) and oligonucleotide CpGmotif (CpG)	[82]

**Table 3:** Selected clinical studies involving liposomal carrier system.

Condition	Sponsor/Collaborators	Status
Lung cancer	Eastern Cooperative Oncology Grp.; National Cancer Institute (NCI)	Phase II
Chronic Lymphocytic Leukemia (CLL)	XEME biopharma Inc.; National Cancer Institute (NCI)	Phase I
Tuberculosis	National Institute of Allergy and Infectious Disease (NIAID)	Phase I
Non-small cell lung cancer	EMD Serono; Merck KGaA	Phase III
Breast cancer	EMD Serono	Phase III
Influenza	NasVax Ltd.	Phase II
Tuberculosis	Statens Serum Institut	Phase I

**Table 4:** Modifications of PLGA nanoparticles for vaccine delivery.

Disease	Antigen	Immuno-Adjuvant	Modification	Ref.
Cancer (Melanoma)	Melan-A:26 , gp100:209 (peptides). OVA as model antigen.	Poly (I:C), CpG	Mannose functionalized delivery system (PLGA, PEG-PLGA and Mannose-PEG-PCL) was developed to target CD206/MR on DC.	[96]
	MART-1 (peptide)	-	Biotinylated anti-human DEC-205 monoclonal antibodies were used to target DCs.	[97]
Cancer	OVA as model protein antigen	Pam3Csk4, Poly (I:C)	Agonistic α-CD40-mAb were conjugated on the surface of PLGA nanoparticles for CD-40 targeted DC delivery.	[98]
	Cancer cell membrane obtained from mouse-melanoma cells	-	PLGA nanoparticle were coated with cancer cell membrane to introduce multiple surface antigen which is challenging with traditional synthetic methods.	[99]
	OVA as model protein antigen along with SOCS1 siRNA	-	Silencing of immunosuppressive SOCS1 gene augmented pro-inflammatory cytokine response.	[100]
Improved Hybrid polymer-lipid particle	BSA	-	Cholesterol coated PLGA particle showed improved stability with better cellular uptake and more controlled antigen release.	[101]
Malaria	Pfs25 (*Plasmodium falciparum* Transmission-Blocking Antigen)	-	-	[102]
	VMP001	MPLA	Lipid (DOPC, DOPG, mal-PE) coated PLGA particles were developed with surface presentation of antigen using maleimide-thiol conjugation.	[103]

## Abbreviations

The following abbreviations are used in this manuscript:

ALF	Army Liposome Formulation
CD 80	Cluster of Differentiation 80
CFA	Complete Freund’s adjuvant
DDA	Dimethyl dioctadecyl-ammonium bromide
DLPE	1,2-dilauroyl-sn-glycero-3-phosphoethanolamine
DMG	1,2-Dimyristoyl-sn-glycerol
DMPC	Dimyristoyl phosphatidylcholine
DMPG	Dimyristoyl phosphatidylglycerol
DOPC	1,2-dioleoyl-sn-glycero-3-phosphocholine
DOPE	1,2-dioleoyl-sn-glycero-3-phosphoethanolamine
DOPG	1,2-dioleoyl-sn-glycero-3-phoshpo-(1’-rac-gylcerol)
DOTAP	1,2-dioleoyl-3-trimethylammonium propane
DPPC	Dipalmitoyl phosphatidylcholine
DSPE-PEG	1,2-distearoyl-sn-glycero-3-phosphoethanolamine-N-[methoxy (polyethyleneglycol)]
EPC	Egg phosphatidylcholine
HBsAg	Recombinant human hepatitis B virus surface antigen
IFA	Incomplete Freund’s adjuvant
INF-γ	Interferon γ
IVTT	*in vitro* transcription and translation
MART-1	Melanoma antigen recognized by T-cells 1
MHC	Major Histocompatibility Complex
MPC	Mannose-PEG1000-cholesterol
MPER	Membrane proximal external region
MPLA	Monophosphoryl lipid A
NK cells	Natural Killer cells
PC	Phosphatidylcholine
PEG	Polyethylene Glycol
POPE	1-palmitoyl-2-oleoyl-sn-glycero-3-phosphoethanolamine
POPG	1-palmitoyl-2-oleoyl-sn-glycero-3-phosphatidylglycerol
SA	Stearyl amine
S1P	Sphingosine-1-phosphate
SPC	Soy phosphatidylcholine
TDB	d-(+)-trehalose 6,6-dibehenate
VMP	Vivax malaria protein

## References

[1] Ragupathi G., Damani P., Srivastava G., Srivastava O., Sucheck S.J., Ichikawa Y., Livingston P.O. (2009). Synthesis of sialyl Lewis^a^ (sLe^a^, CA19-9) and construction of an immunogenic sLe^a^ vaccine. Cancer Immunol. Immunother..

[2] Zhu J., Warren J.D., Danishefsky S.J. (2009). Synthetic carbohydrate-based anticancer vaccines: The memorial sloan-kettering experience. Expert Rev. Vaccines.

[3] da Costa C., Walker B., Bonavia A. (2015). Tuberculosis vaccines—State of the art, and novel approaches to vaccine development. Int. J. Infect. Dis..

[4] Fletcher H.A. (2015). Profiling the host immune response to tuberculosis vaccines. Vaccine.

[5] Tam P.H., Lowary T.L. (2010). Mycobacterial lipoarabinomannan fragments as haptens for potential anti-tuberculosis vaccines. Carbohydrate Chemistry: Chemical and Biological Approaches.

[6] Baxter D. (2007). Active and passive immunity, vaccine types, excipients and licensing. Occup. Med..

[7] Scott C. (2004). Classifying vaccines: From cowpox to the cutting edge. BioProcess Int..

[8] Datta J., Terhune J.H., Lowenfeld L., Cintolo J.A., Xu S., Roses R.E., Czerniecki B.J. (2014). Optimizing dendritic cell-based approaches for cancer immunotherapy. Yale J. Biol. Med..

[9] Amigorena S., Savina A. (2010). Intracellular mechanisms of antigen cross presentation in dendritic cells. Curr. Opin. Immunol..

[10] Alexopoulou L., Holt A.C., Medzhitov R., Flavell R.A. (2001). Recognition of double-stranded RNA and activation of NF-κB by Toll-like receptor 3. Nature.

[11] Janeway C.A., Medzhitov R. (2002). Innate immune recognition. Annu. Rev. Immunol..

[12] Danishefsky S.J., Allen J.R. (2000). From the laboratory to the clinic: A retrospective on fully synthetic carbohydrate-based anticancer vaccines. Angew. Chem. Int. Ed..

[13] Ouerfelli O., Warren J.D., Wilson R.M., Danishefsky S.J. (2005). Synthetic carbohydrate-based antitumor vaccines: Challenges and opportunities. Expert Rev. Vaccines.

[14] Glunz P.W., Hintermann S., Schwarz J.B., Kuduk S.D., Chen X.T., Williams L.J., Sames D., Danishefsky S.J., Kudryashov V., Lloyd K.O. (1999). Probing cell surface “glyco-architecture” through total synthesis. Immunological consequences of a human blood group determinant in a clustered mucin-like context. J. Am. Chem. Soc..

[15] Glunz P.W., Hintermann S., Williams L.J., Schwarz J.B., Kuduk S.D., Kudryashov V., Lloyd K.O., Danishefsky S.J. (2000). Design and synthesis of Ley-bearing glycopeptides that mimic cell surface Ley mucin glycoprotein architecture. J. Am. Chem. Soc..

[16] Kudryashov V., Glunz P.W., Williams L.J., Hintermann S., Danishefsky S.J., Lloyd K.O. (2001). Toward optimized carbohydrate-based anticancer vaccines: Epitope clustering, carrier structure, and adjuvant all influence antibody responses to Lewisy conjugates in mice. Proc. Natl. Acad. Sci. USA.

[17] Toyokuni T., Dean B., Cai S., Boivin D., Hakomori S., Singhal A.K. (1994). Synthetic vaccines: Synthesis of a dimeric Tn antigen-lipopeptide conjugate that elicits immune responses against Tn-expressing glycoproteins. J. Am. Chem. Soc..

[18] Toyokuni T., Hakomori S.I., Singhal A.K. (1994). Synthetic carbohydrate vaccines: Synthesis and immunogenicity of Tn antigen conjugates. Bioorg. Med. Chem..

[19] Buskas T., Ingale S., Boons G.J. (2005). Towards a fully synthetic carbohydrate-based anticancer vaccine: Synthesis and immunological evaluation of a lipidated glycopeptide containing the tumor-associated Tn antigen. Angew. Chem. Int. Ed..

[20] Krikorian D., Panou-Pomonis E., Voitharou C., Sakarellos C., Sakarellos-Daitsiotis M. (2005). A peptide carrier with a built-in vaccine adjuvant: Construction of immunogenic conjugates. Bioconjugate Chem..

[21] Brocke C., Kunz H. (2002). Synthesis of tumor-associated glycopeptide antigens. Bioorg. Med. Chem..

[22] Buskas T., Thompson P., Boons G.J. (2009). Immunotherapy for cancer: Synthetic carbohydrate-based vaccines. Chem. Commun..

[23] Hanisch F.G. Immunology of MUC1 and Advancements in the Development of MUC1 Glycopeptide Tumor Vaccines: An Update. http://www.eurekaselect.com/117190/chapter/immunology-of-muc1-and-advancements-in-the-development-of-muc1-glycopeptide-tumor-vaccines%3A-an-updat.

[24] Heimburg-Molinaro J., Lum M., Vijay G., Jain M., Almogren A., Rittenhouse-Olson K. (2011). Cancer vaccines and carbohydrate epitopes. Vaccine.

[25] Morelli L., Poletti L., Lay L. (2011). Carbohydrates and immunology: Synthetic oligosaccharide antigens for vaccine formulation. Eur. J. Org. Chem..

[26] Wang L.X. (2013). Synthetic carbohydrate antigens for HIV vaccine design. Curr. Opin. Chem. Biol..

[27] Bernardi A., Jiménez-Barbero J., Casnati A., De Castro C., Darbre T., Fieschi F., Finne J., Funken H., Jaeger K.E., Lahmann M. (2013). Multivalent glycoconjugates as anti-pathogenic agents. Chem. Soc. Rev..

[28] Azuma I., Seya T. (2001). Development of immunoadjuvants for immunotherapy of cancer. Int. Immunopharmacol..

[29] Dredge K., Marriott J.B., Todryk S.M., Dalgleish A.G. (2002). Adjuvants and the promotion of Th1-type cytokines in tumour immunotherapy. Cancer Immunol. Immunother..

[30] Tsan M.F. (2006). Toll-like receptors, inflammation and cancer. Semin. Cancer Biol..

[31] Vogel F.R. (1995). Immunologic adjuvants for modern vaccine formulations. Ann. N. Y. Acad. Sci..

[32] Gregory A.E., Titball R., Williamson D. (2013). Vaccine delivery using nanoparticles. Front. Cell. Infect. Microbiol..

[33] Krishnamachari Y., Geary S.M., Lemke C.D., Salem A.K. (2011). Nanoparticle delivery systems in cancer vaccines. Pharmaceut. Res..

[34] Sahdev P., Ochyl L.J., Moon J.J. (2014). Biomaterials for nanoparticle vaccine delivery systems. Pharmaceut. Res..

[35] Zhao L., Seth A., Wibowo N., Zhao C.X., Mitter N., Yu C., Middelberg A.P. (2014). Nanoparticle vaccines. Vaccine.

[36] Henriksen-Lacey M., Korsholm K.S., Andersen P., Perrie Y., Christensen D. (2011). Liposomal vaccine delivery systems. Expert Opin. Drug Deliv..

[37] Perrie Y., Crofts F., Devitt A., Griffiths H.R., Kastner E., Nadella V. (2016). Designing liposomal adjuvants for the next generation of vaccines. Adv. Drug Deliv. Rev..

[38] Schwendener R.A. (2014). Liposomes as vaccine delivery systems: A review of the recent advances. Ther. Adv. Vaccines.

[39] Shashi K., Satinder K., Bharat P. (2012). A complete review on: Liposomes. Int. J. Pharm..

[40] Watson D.S., Endsley A.N., Huang L. (2012). Design considerations for liposomal vaccines: Influence of formulation parameters on antibody and cell-mediated immune responses to liposome associated antigens. Vaccine.

[41] Sharma A., Sharma U.S. (1997). Liposomes in drug delivery: Progress and limitations. Int. J. Pharm..

[42] Shahum E., Thérien H.M. (1994). Correlation between *in vitro* and *in vivo* behaviour of liposomal antigens. Vaccine.

[43] Tan L., Weisslg V., Gregorladls G. (2011). Comparison of the immune response against polio peptides covalently-surface-linked to and internally-entrapped in liposomes. Asian Pac. J. Allergy Immunol..

[44] Thérien H.M., Lair D., Shahum E. (1990). Liposomal vaccine: Influence of antigen association on the kinetics of the humoral response. Vaccine.

[45] Vannier W., Snyder S. (1988). Antibody responses to liposome-associated antigen. Immunol. Lett..

[46] White W.I., Cassatt D.R., Madsen J., Burke S.J., Woods R.M., Wassef N.M., Alving C.R., Koenig S. (1995). Antibody and cytotoxic T-lymphocyte responses to a single liposome-associated peptide antigen. Vaccine.

[47] Conwell C.C., Huang L. (2005). Recent advances in non-viral gene delivery. Adv. Genet..

[48] Gao X., Huang L. (1995). Cationic liposome-mediated gene transfer. Gene Ther..

[49] Li W., Szoka F. (2007). Lipid-based nanoparticles for nucleic acid delivery. Pharm. Res..

[50] Xu Y., Szoka F.C. (1996). Mechanism of DNA release from cationic liposome/DNA complexes used in cell transfection. Biochemistry.

[51] Akbarzadeh A., Rezaei-Sadabady R., Davaran S., Joo S.W., Zarghami N., Hanifehpour Y., Samiei M., Kouhi M., Nejati-Koshki K. (2013). Liposome: Classification, preparation, and applications. Nanoscale Res. Lett..

[52] Huang Z., Li X., Zhang T., Song Y., She Z., Li J., Deng Y. (2014). Progress involving new techniques for liposome preparation. Asian J. Pharm. Sci..

[53] Patil Y.P., Jadhav S. (2014). Novel methods for liposome preparation. Chem. Phys. Lipids.

[54] Szoka F., Papahadjopoulos D. (1980). Comparative properties and methods of preparation of lipid vesicles (liposomes). Annu. Rev. Biophys. Bioeng..

[55] Manolova V., Flace A., Bauer M., Schwarz K., Saudan P., Bachmann M.F. (2008). Nanoparticles target distinct dendritic cell populations according to their size. Eur. J. Immunol..

[56] Mann J.F., Shakir E., Carter K.C., Mullen A.B., Alexander J., Ferro V.A. (2009). Lipid vesicle size of an oral influenza vaccine delivery vehicle influences the Th1/Th2 bias in the immune response and protection against infection. Vaccine.

[57] Miyabe H., Hyodo M., Nakamura T., Sato Y., Hayakawa Y., Harashima H. (2014). A new adjuvant delivery system ‘cyclic di-GMP/YSK05 liposome’ for cancer immunotherapy. J. Control. Release.

[58] Badiee A., Jaafari M.R., Khamesipour A., Samiei A., Soroush D., Kheiri M.T., Barkhordari F., McMaster W.R., Mahboudi F. (2009). Enhancement of immune response and protection in BALB/c mice immunized with liposomal recombinant major surface glycoprotein of Leishmania (rgp63): The role of bilayer composition. Colloids Surf. B. Biointerfaces.

[59] Garnier F., Forquet F., Bertolino P., Gerlier D. (1991). Enhancement of *in vivo* and *in vitro* T cell response against measles virus haemagglutinin after its incorporation into liposomes: Effect of the phospholipid composition. Vaccine.

[60] Kahl L., Scott C.A., Lelchuk R., Gregoriadis G., Liew F.Y. (1989). Vaccination against murine cutaneous leishmaniasis by using Leishmania major antigen/liposomes. Optimization and assessment of the requirement for intravenous immunization. J. Immunol..

[61] Kersten G., van de Put A.M., Teerlink T., Beuvery E.C., Crommelin D.J. (1988). Immunogenicity of liposomes and iscoms containing the major outer membrane protein of Neisseria gonorrhoeae: Influence of protein content and liposomal bilayer composition. Infect Immun..

[62] Mazumdar T., Anam K., Ali N. (2005). Influence of phospholipid composition on the adjuvanticity and protective efficacy of liposome-encapsulated Leishmania donovani antigens. J. Parasitol..

[63] Christensen D., Korsholm K.S., Andersen P., Agger E.M. (2011). Cationic liposomes as vaccine adjuvants. Expert Rev. Vaccines.

[64] Soema P.C., Willems G.J., Jiskoot W., Amorij J.P., Kersten G.F. (2015). Predicting the influence of liposomal lipid composition on liposome size, zeta potential and liposome-induced dendritic cell maturation using a design of experiments approach. Eur. J. Pharm. Biopharm..

[65] Hafez I., Maurer N., Cullis P. (2001). On the mechanism whereby cationic lipids promote intracellular delivery of polynucleic acids. Gene Ther..

[66] Fan Y., Sahdev P., Ochyl L.J., Akerberg J.J., Moon J.J. (2015). Cationic liposome–hyaluronic acid hybrid nanoparticles for intranasal vaccination with subunit antigens. J. Control. Release.

[67] Karmakar P., Lee K., Sarkar S., Wall K.A., Sucheck S.J. (2016). Synthesis of a liposomal MUC1 glycopeptide-based immunotherapeutic and evaluation of the effect of l-Rhamnose targeting on cellular immune responses. Bioconjugate Chem..

[68] Sarkar S., Salyer A.C., Wall K.A., Sucheck S.J. (2013). Synthesis and immunological evaluation of a MUC1 glycopeptide incorporated into l-rhamnose displaying liposomes. Bioconjugate Chem..

[69] Sarkar S., Lombardo S.A., Herner D.N., Talan R.S., Wall K.A., Sucheck S.J. (2010). Synthesis of a single-molecule L-rhamnose-containing three-component vaccine and evaluation of antigenicity in the presence of anti-L-rhamnose antibodies. J. Am. Chem. Soc..

[70] Oyelaran O., McShane L.M., Dodd L., Gildersleeve J.C. (2009). Profiling human serum antibodies with a carbohydrate antigen microarray. J. Proteome Res..

[71] Jiang P.L., Lin H.J., Wang H.W., Tsai W.Y., Lin S.F., Chien M.Y., Liang P.H., Huang Y.Y., Liu D.Z. (2015). Galactosylated liposome as a dendritic cell-targeted mucosal vaccine for inducing protective anti-tumor immunity. Acta Biomater..

[72] Wang T., Zhen Y., Ma X., Wei B., Li S., Wang N. (2015). Mannosylated and lipid A-incorporating cationic liposomes constituting microneedle arrays as an effective oral mucosal HBV vaccine applicable in the controlled temperature chain. Colloids Surf. B Biointerfaces.

[73] Derrick S.C., Yang A., Parra M., Kolibab K., Morris S.L. (2015). Effect of cationic liposomes on BCG trafficking and vaccine-induced immune responses following a subcutaneous immunization in mice. Vaccine.

[74] Siddiqui K.F., Amir M., Khan N., Rama Krishna G., Sheikh J.A., Rajagopal K., Agrewala J.N. (2015). Prime-boost vaccination strategy with bacillus Calmette-Guerin (BCG) and liposomized alpha-crystalline protein 1 reinvigorates BCG potency. Clin. Exp. Immunol..

[75] Beck Z., Matyas G.R., Jalah R., Rao M., Polonis V.R., Alving C.R. (2015). Differential immune responses to HIV-1 envelope protein induced by liposomal adjuvant formulations containing monophosphoryl lipid A with or without QS21. Vaccine.

[76] Hanson M.C., Abraham W., Crespo M.P., Chen S.H., Liu H., Szeto G.L., Kim M., Reinherz E.L., Irvine D.J. (2015). Liposomal vaccines incorporating molecular adjuvants and intrastructural T-cell help promote the immunogenicity of HIV membrane-proximal external region peptides. Vaccine.

[77] Neumann S., Young K., Compton B., Anderson R., Painter G., Hook S. (2015). Synthetic TRP2 long-peptide and alpha-galactosylceramide formulated into cationic liposomes elicit CD8(+) T-cell responses and prevent tumour progression. Vaccine.

[78] Yuba E., Kanda Y., Yoshizaki Y., Teranishi R., Harada A., Sugiura K., Izawa T., Yamate J., Sakaguchi N., Koiwai K. (2015). pH-sensitive polymer-liposome-based antigen delivery systems potentiated with interferon-gamma gene lipoplex for efficient cancer immunotherapy. Biomaterials.

[79] Nakamura T., Yamazaki D., Yamauchi J., Harashima H. (2013). The nanoparticulation by octaarginine-modified liposome improves α-galactosylceramide-mediated antitumor therapy via systemic administration. J. Control. Release.

[80] Lanzi A., Fehres C.M., de Gruijl T.D., van Kooyk Y., Mastrobattista E. (2014). Effects of antigen-expressing immunostimulatory liposomes on chemotaxis and maturation of dendritic cells *in Vitro* and in human skin explants. Pharmaceut. Res..

[81] Carrera I., Etcheverría I., Fernández-Novoa L., Lombardi V.R., Lakshmana M.K., Cacabelos R., Vigo C. (2015). A Comparative Evaluation of a Novel Vaccine in APP/PS1 Mouse Models of Alzheimer’s Disease. BioMed Res. Int..

[82] Saravanan P., Sreenivasa B.P., Selvan R.P., Basagoudanavar S.H., Hosamani M., Reddy N.D., Nathanielsz J., Derozier C., Venkataramanan R. (2015). Protective immune response to liposome adjuvanted high potency foot-and-mouth disease vaccine in Indian cattle. Vaccine.

[83] Bolhassani A., Javanzad S., Saleh T., Hashemi M., Aghasadeghi M.R., Sadat S.M. (2014). Polymeric nanoparticles: Potent vectors for vaccine delivery targeting cancer and infectious diseases. Hum. Vaccines Immunother..

[84] De Souza Rebouças J., Esparza I., Ferrer M., Sanz M.L., Irache J.M., Gamazo C. (2012). Nanoparticulate adjuvants and delivery systems for allergen immunotherapy. BioMed Res. Int..

[85] Hadinoto K., Sundaresan A., Cheow W.S. (2013). Lipid–polymer hybrid nanoparticles as a new generation therapeutic delivery platform: A review. Eur. J. Pharm. Biopharm..

[86] Kunugi S., Yamaoka T. (2012). Polymers in Nanomedicine.

[87] Morachis J.M., Mahmoud E.A., Almutairi A. (2012). Physical and chemical strategies for therapeutic delivery by using polymeric nanoparticles. Pharmacol. Rev..

[88] Yue H., Ma G. (2015). Polymeric micro/nanoparticles: Particle design and potential vaccine delivery applications. Vaccine.

[89] Silva A.L., Rosalia R.A., Varypataki E., Sibuea S., Ossendorp F., Jiskoot W. (2015). Poly-(lactic-co-glycolic-acid)-based particulate vaccines: Particle uptake by dendritic cells is a key parameter for immune activation. Vaccine.

[90] Rahimian S., Fransen M.F., Kleinovink J.W., Christensen J.R., Amidi M., Hennink W.E., Ossendorp F. (2015). Polymeric nanoparticles for co-delivery of synthetic long peptide antigen and poly IC as therapeutic cancer vaccine formulation. J. Control. Release.

[91] Kumar S., Anselmo A.C., Banerjee A., Zakrewsky M., Mitragotri S. (2015). Shape and size-dependent immune response to antigen-carrying nanoparticles. J. Control. Release.

[92] Mueller S.N., Tian S., DeSimone J.M. (2015). Rapid and persistent delivery of antigen by lymph node targeting PRINT nanoparticle vaccine carrier to promote humoral immunity. Mol. Pharmacol..

[93] Tan J., Shah S., Thomas A., Ou-Yang H.D., Liu Y. (2013). The influence of size, shape and vessel geometry on nanoparticle distribution. Microfluid. Nanofluid..

[94] Anselmo A.C., Zhang M., Kumar S., Vogus D.R., Menegatti S., Helgeson M.E., Mitragotri S. (2015). Elasticity of nanoparticles influences their blood circulation, phagocytosis, endocytosis, and targeting. ACS Nano.

[95] Shima F., Akagi T., Akashi M. (2014). The role of hydrophobicity in the disruption of erythrocyte membrane by nanoparticles composed of hydrophobically modified poly(gamma-glutamic acid). J. Biomater. Sci. Polym. Ed..

[96] Silva J.M., Zupancic E., Vandermeulen G., Oliveira V.G., Salgado A., Videira M., Gaspar M., Graca L., Préat V., Florindo H.F. (2015). *In vivo* delivery of peptides and Toll-like receptor ligands by mannose-functionalized polymeric nanoparticles induces prophylactic and therapeutic anti-tumor immune responses in a melanoma model. J. Control. Release.

[97] Saluja S.S., Hanlon D.J., Sharp F.A., Hong E., Khalil D., Robinson E., Tigelaar R., Fahmy T.M., Edelson R.L. (2014). Targeting human dendritic cells via DEC-205 using PLGA nanoparticles leads to enhanced cross-presentation of a melanoma-associated antigen. Int. J. Nanomedicine.

[98] Rosalia R.A., Cruz L.J., van Duikeren S., Tromp A.T., Silva A.L., Jiskoot W., de Gruijl T., Löwik C., Oostendorp J., van der Burg S.H. (2015). CD40-targeted dendritic cell delivery of PLGA-nanoparticle vaccines induce potent anti-tumor responses. Biomaterials.

[99] Fang R.H., Hu C.M., Luk B.T., Gao W., Copp J.A., Tai Y., O’Connor D.E., Zhang L. (2014). Cancer cell membrane-coated nanoparticles for anticancer vaccination and drug delivery. Nano Lett..

[100] Heo M.B., Cho M.Y., Lim Y.T. (2014). Polymer nanoparticles for enhanced immune response: Combined delivery of tumor antigen and small interference RNA for immunosuppressive gene to dendritic cells. Acta Biomater..

[101] Hu Y., Hoerle R., Ehrich M., Zhang C. (2015). Engineering the lipid layer of lipid-PLGA hybrid nanoparticles for enhanced *in vitro* cellular uptake and improved stability. Acta Biomater..

[102] Kumar R., Ledet G., Graves R., Datta D., Robinson S., Bansal G.P., Mandal T., Kumar N. (2015). Potent functional immunogenicity of plasmodium falciparum transmission-blocking antigen (Pfs25) delivered with nanoemulsion and porous polymeric nanoparticles. Pharmaceut. Res..

[103] Moon J.J., Suh H., Polhemus M.E., Ockenhouse C.F., Yadava A., Irvine D.J. (2012). Antigen-displaying lipid-enveloped PLGA nanoparticles as delivery agents for a Plasmodium vivax malaria vaccine. PLoS ONE.

[104] Orozco V.H., Palacio J., Sierra J., López B.L. (2013). Increased covalent conjugation of a model antigen to poly(lactic acid)-g-maleic anhydride nanoparticles compared to bare poly(lactic acid) nanoparticles. Colloid Polym. Sci..

[105] Rahimian S., Kleinovink J.W., Fransen M.F., Mezzanotte L., Gold H., Wisse P., Overkleeft H., Amidi M., Jiskoot W., Löwik C.W. (2015). Near-infrared labeled, ovalbumin loaded polymeric nanoparticles based on a hydrophilic polyester as model vaccine: *In vivo* tracking and evaluation of antigen-specific CD8(+) T cell immune response. Biomaterials.

[106] Kunda N.K., Alfagih I.M., Miyaji E.N., Figueiredo D.B., Gonçalves V.M., Ferreira D.M., Dennison S.R., Somavarapu S., Hutcheon G.A., Saleem I.Y. (2015). Pulmonary dry powder vaccine of pneumococcal antigen loaded nanoparticles. Int. J. Pharm..

[107] Zhang P., Chiu Y.C., Tostanoski L.H., Jewell C.M. (2015). Polyelectrolyte multilayers assembled entirely from immune signals on gold nanoparticle templates promote antigen-specific T cell response. ACS Nano.

[108] Chiu Y.C., Gammon J.M., Andorko J.I., Tostanoski L.H., Jewell C.M. (2015). Modular vaccine design using carrier-free capsules assembled from polyionic immune signals. ACS Biomater. Sci. Eng..

[109] Chiodo F., Enríquez-Navas P.M., Angulo J., Marradi M., Penadés S. (2015). Assembling different antennas of the gp120 high mannose-type glycans on gold nanoparticles provides superior binding to the anti-HIV antibody 2G12 than the individual antennas. Carbohydr. Res..

[110] Almeida J.P., Lin A.Y., Figueroa E.R., Foster A.E., Drezek R.A. (2015). *In vivo* gold nanoparticle delivery of peptide vaccine induces anti-tumor immune response in prophylactic and therapeutic tumor models. Small.

[111] Biswas S., Medina S.H., Barchi J.J. (2015). Synthesis and cell-selective antitumor properties of amino acid conjugated tumor-associated carbohydrate antigen-coated gold nanoparticles. Carbohydr. Res..

[112] Mocan T., Matea C., Tabaran F., Iancu C., Orasan R., Mocan L. (2015). *In vitro* administration of gold nanoparticles functionalized with MUC-1 protein fragment generates anticancer vaccine response via macrophage activation and polarization mechanism. J. Cancer.

[113] Tavernaro I., Hartmann S., Sommer L., Hausmann H., Rohner C., Ruehl M., Hoffmann-Roeder A., Schlecht S. (2015). Synthesis of tumor-associated MUC1-glycopeptides and their multivalent presentation by functionalized gold colloids. Org. Biomol. Chem..

[114] Tao W., Gill H.S. (2015). M2e-immobilized gold nanoparticles as influenza a vaccine: Role of soluble M2e and longevity of protection. Vaccine.

[115] Kumar R., Ray P.C., Datta D., Bansal G.P., Angov E., Kumar N. (2015). Nanovaccines for malaria using plasmodium falciparum antigen Pfs25 attached gold nanoparticles. Vaccine.

[116] Dykman L.A., Staroverov S.A., Mezhenny P.V., Fomin A.S., Kozlov S.V., Volkov A.A., Laskavy V.N., Shchyogolev S.Y. (2015). Use of a synthetic foot-and-mouth disease virus peptide conjugated to gold nanoparticles for enhancing immunological response. Gold Bull..

[117] Gianvincenzo P.D., Calvo J., Perez S., Álvarez A., Bedoya L.M., Alcamí J., Penadés S. (2015). Negatively charged glyconanoparticles modulate and stabilize the secondary structures of a gp120 V3 loop peptide: Toward fully synthetic HIV vaccine candidates. Bioconjugate Chem..

[118] Sungsuwan S., Yin Z., Huang X. (2015). Lipopeptide-coated iron oxide nanoparticles as potential glycoconjugate-based synthetic anticancer vaccines. ACS Appl. Mater. Interfaces.

[119] Ramella D., Polito L., Mazzini S., Ronchi S., Scaglioni L., Marelli M., Lay L. (2014). A strategy for multivalent presentation of carba analogues from *N. meningitidis* a capsular polysaccharide. Eur. J. Org. Chem..

[120] den Brok M.H., Sutmuller R.P., van der Voort R., Bennink E.J., Figdor C.G., Ruers T.J., Adema G.J. (2004). *In situ* tumor ablation creates an antigen source for the generation of antitumor immunity. Cancer Res..

[121] O’Neal D.P., Hirsch L.R., Halas N.J., Payne J.D., West J.L. (2004). Photo-thermal tumor ablation in mice using near infrared-absorbing nanoparticles. Cancer Lett..

[122] Ito A., Kobayashi T. (2008). Intracellular hyperthermia using magnetic nanoparticles: A novel method for hyperthermia clinical applications. Therm. Med..

[123] Kobayashi T. (2011). Cancer hyperthermia using magnetic nanoparticles. Biotech. J..

